# Breast Milk Cytokines and Early Growth in Gambian Infants

**DOI:** 10.3389/fped.2018.00414

**Published:** 2019-01-17

**Authors:** Anja Saso, Oleg Blyuss, Daniel Munblit, Amadou Faal, Sophie E. Moore, Kirsty Le Doare

**Affiliations:** ^1^Department of Paediatrics, Imperial College London, London, United Kingdom; ^2^MRC Unit The Gambia at the London School of Hygiene and Tropical Medicine, Banjul, Gambia; ^3^Centre for Cancer Prevention, Wolfson Institute of Preventative Medicine, Queen Mary University of London, London, United Kingdom; ^4^Department of Women's Cancer, Institute for Women's Health, University College London, London, United Kingdom; ^5^N. I. Lobachevsky State University of Nizhny Novgorod, Nizhny Novgorod, Russia; ^6^Solov'ev Research and Clinical Center for Neuropsychiatry, Moscow, Russia; ^7^Department of Paediatrics, Sechenov University, Moscow, Russia; ^8^Department of Women and Children's Health, King's College London, London, United Kingdom; ^9^Centre for International Child Health, Imperial College London, London, United Kingdom; ^10^West Africa Global Health Alliance, Banjul, Gambia; ^11^Paediatric Infectious Diseases Research Group, St George's University of London, London, United Kingdom

**Keywords:** breast milk, colostrum, cytokine, growth, weight, infant, neonate, immunity

## Abstract

**Background:** Breast milk provides nutrition for infants but also delivers other bioactive factors that have key protective and developmental benefits. In particular, cytokines are thought to play a role in immunomodulation, although little is known about their impact on health outcomes in early life.

**Objective:** The purpose of this pilot study was to evaluate the relationship between cytokines in breast milk and infant growth outcomes in a low-income setting.

**Methods:** 100 mother-infant pairs were followed up to 2–3 months postpartum as part of a prospective longitudinal cohort study in urban Gambia, West Africa. The concentrations of 9 pro-inflammatory cytokines (IL-1β, IL-2, IL-4, IL-6, IL-10, IL-12, IL-13, IFN-γ, TNFα), IGF-1 and TGFβ2 were measured in colostrum within 12 h of birth and in breast milk at the final visit, scheduled between day 60 and 89 postpartum. Infant weight was recorded and converted to weight-for-age *Z*-scores (WAZ) at the same time points. Growth outcomes were defined in our study as (a) change in WAZ between birth and final visit (b) WAZ at final visit. Linear regression analysis was used to determine the ability of colostrum and breast milk cytokine concentrations to predict growth outcomes up to 2–3 months postpartum.

**Results:** Gambian infants demonstrated growth faltering across the first 2–3 months postpartum. There was no significant relationship between cytokines in colostrum and subsequent change in WAZ between birth and the final visit, in either unadjusted or adjusted models. However, cytokines in mature breast milk, TNFα, IFNγ, IL1β, IL2, IL4, and IL6, were weak negative predictors of WAZ scores at the final visit, in unadjusted models (*p* < 0.05). When adjusted for maternal anemia (as a proxy for maternal nutrition), TNFα and IL6 remained significant predictors (*p* < 0.05).

**Conclusions:** Variations in breast milk cytokine levels do not play a substantial role in the growth faltering observed across early infancy. The potential contribution of other factors, such as micronutrients, hormones or human milk oligosaccharides, must be elucidated. Cytokine levels in mature breast milk were weakly predictive of poor infant growth, possibly reflecting a “read-out” of suboptimal maternal health and nutrition.

## Introduction

Human breast milk (BM) provides nourishment for infants but also delivers a range of non-nutritive bioactive factors that have key protective and developmental benefits, including actively shaping and priming the infant immune system (1–6). It is increasingly recognized that the complex effects of colostrum and BM on the diverse nature and function of the infant microbiome are key to coordinating these roles (7–10). Moreover, biologically active substances influence the infant's response to energy intake and metabolism (3, 11–13). BM composition, however, is very diverse, dynamic, varying between women and over the course of lactation, and sensitive to both maternal and environmental factors (14–16). The exact nature of these variations, their impact on infant morbidity and growth outcomes and the exploitative potential for future interventions has yet to be fully elucidated (10, 17–22).

Cytokines and growth factors have been identified as important bioactive components within BM and are found in differing concentrations (23, 24). It is hypothesized that the mammary gland is the principal source of these compounds in milk (6, 25). In addition, leukocytes isolated from expressed human milk have demonstrated the ability to secrete a small number of cytokines (26). Once ingested, milk-derived cytokines and growth factors pass through the infant's gastrointestinal tract, resisting proteolysis and retaining their bioactivity. They subsequently interact with specific gut epithelial receptors, enabling them to be absorbed from the gastrointestinal tract and thereby enter the infant circulation (12, 18, 19, 27).

Cytokines, particularly in colostrum, are thought to play a key role in immunomodulation, including promoting sIgA production, mediating infant immune responses, for example through B cell growth and differentiation, and boosting development of intestinal tolerance (4, 5, 12, 28, 29). However, the impact of these immune factors on early infant growth and body composition is less well-documented.

Growth faltering in sub-Saharan Africa, including The Gambia, has been well-established previously (30) and poor growth in infancy and early childhood has adverse consequences for child mortality, and on subsequent adult stature and health outcomes (31, 32). At the same time, the rate of breast feeding is high in these low-income populations and is the only sustainable feeding option for many. Despite intensive efforts aimed at achieving optimal nourishment for mother and child, substantial infant growth faltering remains, suggesting the potential importance of other non-nutritive factors (30, 33). A more thorough understanding of how natural variation in BM bioactive components influences infant development and may exacerbate or protect against growth failure would help guide the development of preventative and therapeutic strategies, particularly in low-income settings (21, 31).

The aim of this study was to evaluate the relationship between the cytokine and growth factor profile in human BM and infant growth outcomes in the first 3 months post-partum in The Gambia, a low-income country in West Africa.

## Materials and Methods

### Study Setting, Population, and Ethics

**Figure 1:**
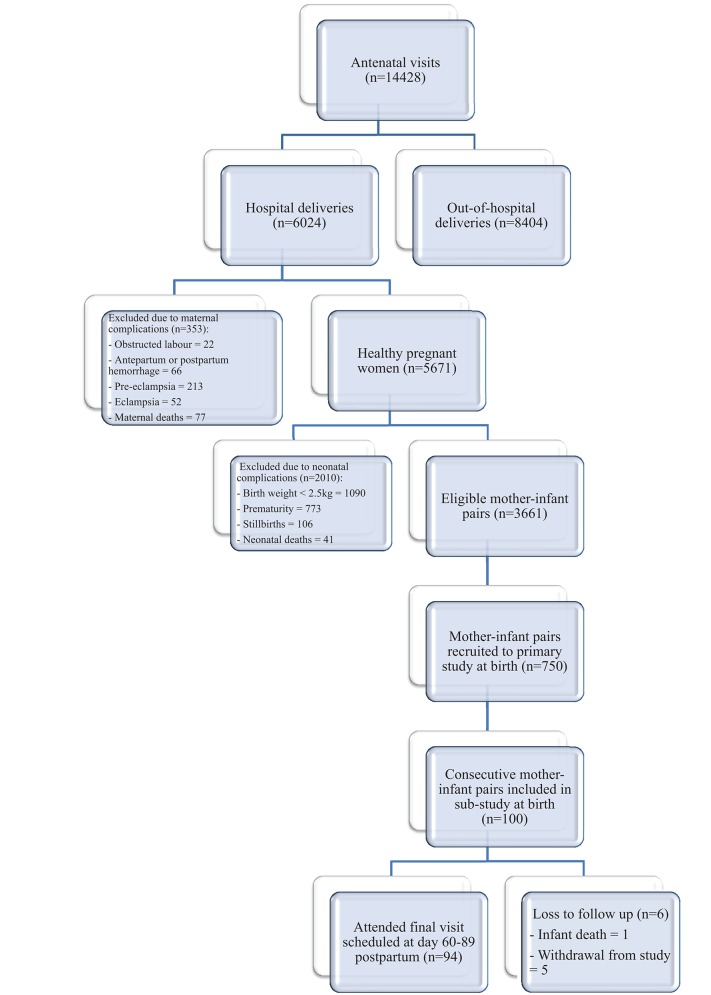
Flow diagram to demonstrate recruitment, retention and loss to follow up (34).

Samples for this project were made available as part of a larger prospective longitudinal cohort study on *Group B Streptococcus* infection, conducted between 1st January and 31st December 2014, in an urban area of The Gambia, West Africa (34). The Gambia is a low-income country with an estimated 79,000 births annually and an infant mortality rate of 42/1,000 (2016) (35). Moreover, 47% of infants are exclusively breastfed under 6 months and 98% breastfed (mixed and exclusive) beyond the first year of life (2013) (35). The cohort in this sub-project consisted of 100 consecutive pregnant women and their infants followed up across the first 2–3 months postpartum in two government health centers offering antenatal care to women. These centers were considered representative samples of the standard of care available to Gambian women in urban settings, with a combined birth rate of ~12,500 births annually(34). Women between the ages of 18–40 years who had a negative HIV test and low-risk, singleton pregnancy (no evidence of pre-eclampsia, cardiomyopathy, maternal gestational diabetes, placenta praevia, twin pregnancy) were recruited in the final trimester of pregnancy after giving informed consent. They were excluded if they did not plan to breastfeed or remain in the nearby area for the first 3 months postpartum. Infant exclusion criteria included marked prematurity (<32 weeks gestation), low birth weight (<2.5 kg), congenital abnormalities or requiring resuscitation at the time of delivery with subsequent transfer to a neonatal unit. Further demographic and recruitment details, including eligibility, inclusion and exclusion criteria and consent protocol, are summarized in Figure 1 and have previously been published (34). The study was approved by the joint Gambian Government/Medical Research Council Research Ethics Committee, SCC 1350 V4.

### Data Collection

Following birth, participants were monitored daily at home until day 6 and then asked to return to the clinic for a final visit which was scheduled between day 60 and 89 postpartum. Details were documented via standardized questionnaires and included maternal factors (age, ethnic origin, gravida, parity, weight, blood pressure, hemoglobin concentration, illnesses in pregnancy, use of antibiotics/medications/ traditional medicines/antimalarial prophylaxis) and infant factors (anthropometry, sex, morbidity, vaccinations, vital signs, feeding). Full details on data collection have been described previously (34). Anthropometric measures were performed by trained field nurses at birth and at the final visit; infant weight was measured using electronic baby scales (model 336; Seca, UK), to the nearest 0.01 kg. Weight-for-age Z score (WAZ) was calculated based on the WHO growth standards (36).

### Colostrum and Breast Milk Sample Collection

Colostrum was collected within 12 h of birth and mature BM was collected at the final visit. After washing their hands with soap, mothers wiped each breast with a 0.02% chlorhexidine wipe and then hand-expressed their milk prior to feeding their infant; the first 0.5 mls was discarded and then 2–3 mL of colostrum and 4–5 mL of BM was collected from both breasts into sterile plastic containers. Milk samples were immediately stored on cold packs at 4°C and transported to the laboratory within 6 h of collection (37, 38).

### Laboratory Procedures

BM samples were centrifuged at 3,200 rpm for 30 min to separate whey and lipid layers. The lipid layer was removed with a scalpel and stored separately. The whey layer was stored in 1 mL aliquots and frozen at −70°C. It was transferred to 5 mm NMR capillary tubes for analysis (37, 38).

### Cytokine Quantification

Assays were run in duplicate and concentrations of IL-1β, IL-2, IL-4, IL-6, IL-10, IL-12, IL-13, IFN-γ, TNFα, IGF-1, and TGFβ2 in colostrum and BM were determined by electroluminescence using kits from Meso-Scale Discovery Multi-Spot Kit (MSD, Rockville, MD, United States). Laboratory experiments were run according to manufacturer's protocol, using an eight-point standard curve. Samples below the lower limit of detection were allocated a concentration for each cytokine of half the lower limit of detection to aid the statistical analysis and as per manufacturer's instructions (Supplementary Table 1). The coefficient of variance (CoV) for the assay was 25% (37, 38).

### Growth Outcomes

Growth outcomes were defined in our study as (a) change in WAZ between birth and final visit b) WAZ at final visit. Children with Z scores below −2 were defined as underweight (WAZ ≤ −2) (36).

### Statistical Analysis

The sample size was based on a sample of convenience for a pilot study of 100 mother-infant pairs as there are no published studies of expected cytokine values in milk and their association with growth outcomes on which to base our calculations. Descriptive statistics were calculated for maternal and infant demographics, infant growth parameters and cytokines in colostrum and mature BM. Shapiro-Wilk test was used to assess the normality of the distribution of cytokines. Because all measurements deviated significantly from normality, non-parametric tests were used in further analysis. Potential differences and associations between cytokines in colostrum and mature BM were calculated using Wilcoxon rank-sum and Spearman rank correlation tests, respectively. Differences in WAZ scores between male and female infants were calculated using Wilcoxon rank sum tests. All other univariate analysis was performed using simple linear regression on log transformed data. Multivariate linear regression (including stepwise regression) analysis was then used to determine the ability of cytokine concentrations to predict longitudinal growth outcomes in the first 2–3 months post-partum by analyzing the association between (i) colostrum cytokines and change in WAZ between birth and final visit (ii) BM cytokines and WAZ at final visit. Potential maternal and infant confounders found to be significant in variable univariate analysis with a *p* < 0.1 or known to be associated with infant growth (*a priori*) were included in the multivariate models. Maternal hemoglobin (Hb) was categorized as either Hb <11 g/dL (anemia) or Hb ≥11 g/dL (39). Maternal illness was categorized as either having none or ≥ 1 self-reported acutely unwell episodes recorded during pregnancy, regardless of severity or outcome. Maternal gravida was categorized as either nil or ≥1 previous pregnancies, regardless of outcome. Maternal parity was defined as either nil or ≥1 previous pregnancies carried to a viable gestational age.

All statistical analyses were completed using the statistical packages STATA version 12 (StataCorp 2013, California CA, United States), GraphPad Prism version 6.0 (GraphPad Softward Inc., La Jolla, CA, United States) and R package version 3.4.1. Differences were considered statistically significant at *p* < 0.05.

## Results

### Maternal and Infant Characteristics

A total of 100 mother-infant pairs were included in the analysis. The mean ± 1 SD maternal age was 25.6 ± 5.7 years and mean ± 1 SD maternal weight at delivery was 63.4 ± 9.8 kg. The infants were born at mean gestational age of 39 weeks and followed up to a mean ± 1 SD of 61.6 ± 1.3 days postpartum; although scheduling allowed for final visits to occur up to 90 days postpartum, all final visits in this cohort took place between 60 and 66 days postpartum. All infants were exclusively breast fed throughout the duration of the study. Demographic characteristics of mother-infant pairs are summarized in Table 1.

**Table 1:** Description of study population.

**Variable**	**Value**
Maternal age, years (mean ± SD)	25.6 ± 5.7
Maternal weight, kg (mean ± SD)	63.4 ± 9.8
Gestational age at birth, weeks (mean ± SD)	39.0 ± 2.5
Length of post-partum follow-up, days (mean ± SD)	61.6 ± 1.3
**MATERNAL Hb CATEGORIES**, ***n*** **(%)**	
Hb ≥ 11g/dL	33 (40)
Hb <11g/dL	49 (60)
**MATERNAL ILLNESS CATEGORIES**, ***n*** **(%)**	
Illness during pregnancy	16 (18)
No illness during pregnancy	73 (82)
**PARITY CATEGORIES**, ***n*** **(%)**	
Primiparous	21 (21)
Multiparous	79 (79)
**GESTATIONAL AGE CATEGORIES**, ***n*** **(%)**	
≥37 weeks	76 (76)
<37 weeks	24 (24)
**INFANT SEX**, ***n*** **(%)**	
Male	50 (50)
Female	50 (50)

*Values are rounded to 1 decimal place*.

*Missing values: maternal weight = 12, maternal Hb = 18, maternal illness = 11*.

*SD, standard deviation; Hb, hemoglobin*.

**Figure 2:**
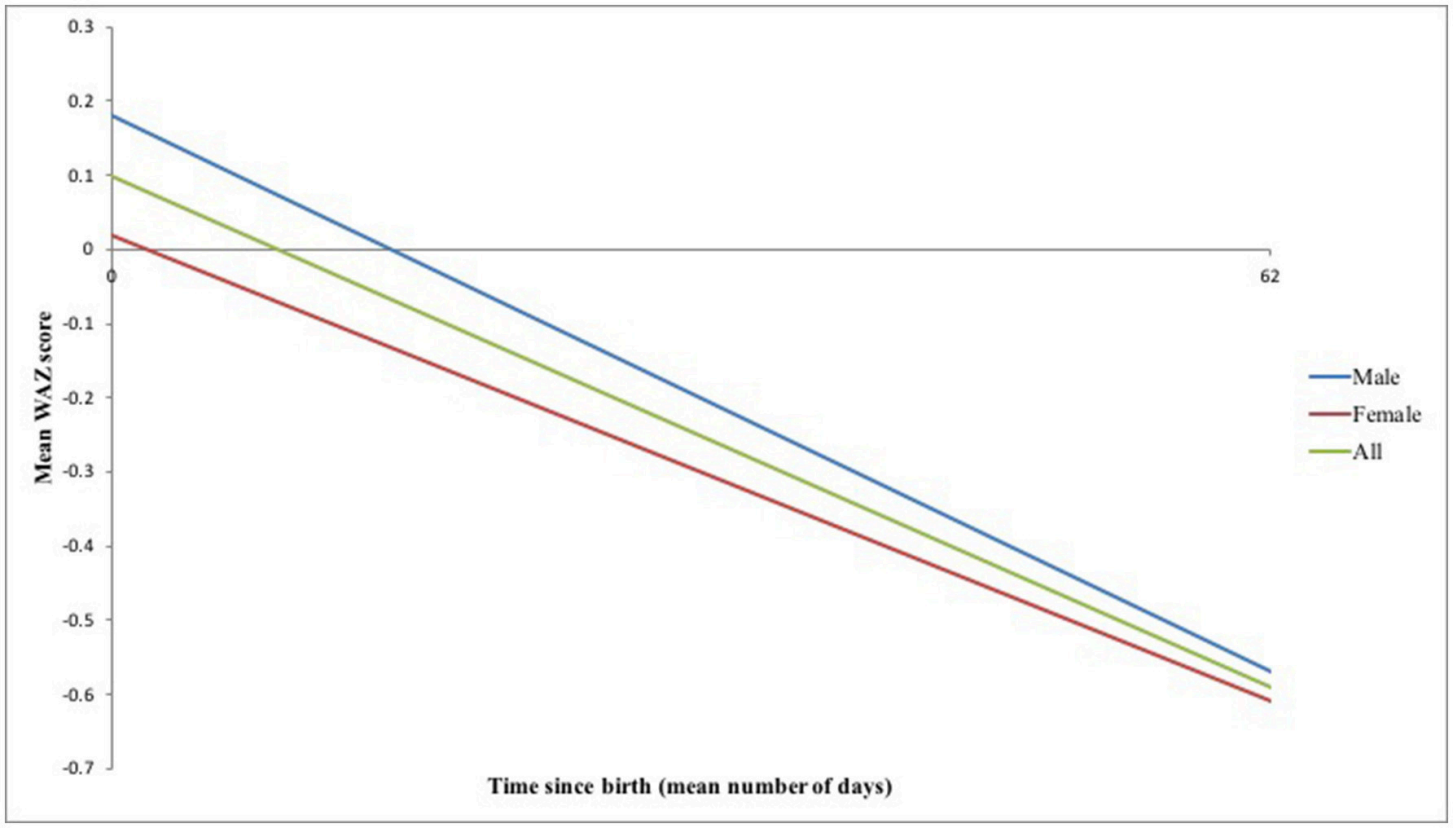
Mean WAZ scores across the first 2–3 months postpartum.

Infants were born with a mean weight ± 1 SD of 3.36 ± 0.48 kg and WAZ ± 1 SD of 0.10 ± 0.99 (Table 2A). However, substantial growth faltering was seen across the first 2–3 months postpartum, equivalent to crossing over one major centile band (Figure 2). Of the 94/100 infants remaining in the study at the final visit, infants' WAZ had declined to a mean ± 1 SD of −0.59 ± 1.20; 71% of infants demonstrated a decline in their WAZ and 15% were underweight by 2–3 months postpartum (WAZ< -2) (Tables 2A,B). The difference between male and female infant WAZ scores was non-significant at birth (*p* = 0.505) and final visit (*p* = 0.791). Similarly, the odds ratio of being underweight and having a decline in WAZ by 2–3 months postpartum did not differ significantly between the sexes: for male infants, the odds ratio was 1.4 (CI 0.45–4.41, *p* = 0.56) and 1.69 (CI 0.68–4.1775, *p* = 0.25), respectively.

**Table 2:** Summary of infant growth data at birth and final visit.

**(A) INFANT WEIGHT AND WAZ SCORES**.		
**Infant growth indicator, mean** **±** **SD**	**Birth**	**Final visit**
**Weight (kg)**		
Male	3.45 ± 0.43	5.24 ± 0.68
Female	3.27 ± 0.42	4.84 ± 0.74
All	3.36 ± 0.48	5.04 ± 0.74
**WAZ score**		
Male	0.18 ± 0.85	−0.57 ± 1.05
Female	0.02 ± 1.12	−0.61 ± 1.34
All	0.10 ± 0.99	−0.59 ± 1.20
**(B) INFANT WAZ CATEGORIES**		
**WAZ categories, n (%)**	**Birth**	**Final visit**
**≤−3**		
Boys	0	0 (0)
Girls	0	3 (100)
All	0	3
**−3** **<** **WAZ** **≤−2**		
Boys	0	8 (72)
Girls	0	3 (27)
All	0	11
**>** **−2**		
Boys	49 (51)	39 (49)
Girls	48 (49)	41 (51)
All	97	80

*All values are rounded to 2 decimal places; WAZ, Weight-for-age Z score; SD, standard deviation*.

### Cytokines in Colostrum and Breast Milk

83 samples of colostrum (from 100 women) and 90 samples of mature breast milk (from 94 women) were successfully collected. In the remaining cases, insufficient sample was available, either because the mother was unable to express milk, or due to insufficient sample remaining after the primary analysis was complete. Colostrum at birth contained significantly higher concentrations of nearly all cytokines than BM (*p* < 0.001) (Supplementary Table 2). Furthermore, the percentage of samples with detectable concentrations was significantly greater for the majority of cytokines in colostrum when compared to BM (*p* < 0.05), with the exception of TNFα, IGF1, TGFβ2, and IL6 (the latter was almost significant, *p* = 0.06). Of note, the levels of IGF1 in colostrum and BM were below the assay limit of detection in >75% of subjects and, therefore, this data has been removed from further analysis (Supplementary Table 2). There were minimal significant associations between the levels of cytokines in colostrum and in mature milk, with only a weak positive correlation found with IL12 (*r* = 0.257, *p* = 0.023) (Supplementary Table 3).

### Associations Between Maternal/Infant Factors and Infant Growth Outcomes

Only maternal illness and infant gestational age at delivery were found to be significant or near significant predictors of change in WAZ between birth and final visit, in univariate analysis (*p* < 0.05) (Table 3). There were no significant maternal or infant predictors of WAZ at final visit.

**Table 3:** Relationship between maternal/infant factors and difference in WAZ at birth and final visit.

**Maternal/infant factor**	**β**	***p-value***
Maternal illness	0.646	0.064
Gestation	0.095	0.077
Maternal age	0.018	0.423
Maternal weight	0.003	0.840
Maternal Hb	−0.200	0.449
Gravida	0.082	0.276
Parity	0.085	0.273
Infant sex	−0.388	0.140

*All values are rounded to 3 decimal places*.

*Linear regression β coefficients for maternal/infant factors predicting difference in WAZ at birth and final visit*.

*WAZ, Weight-for-age Z score; Hb, hemoglobin*.

### Associations Between Cytokines and Infant Growth Outcomes

The association between colostrum cytokines and change in WAZ between birth and final visit was non-significant in both the unadjusted and adjusted models in this Gambian cohort.

Moreover, unadjusted models demonstrated that lower TNFα, IFN-γ, IL1β, IL2, IL4, and IL6 levels in BM were weakly associated with a higher WAZ at final visit (*p* < 0.05). Although significant, the **β** -coefficients were small. When these models were adjusted for maternal anemia, only TNFα and IL6 remained significant predictors of WAZ at final visit (*p* < 0.05) (Table 4). Other multivariate models were non-significant.

**Table 4:** Relationship between breast milk cytokine concentration and WAZ at final visit.

	**Unadjusted analysis**				Adjusted analysis*			
**Breast milk cytokine**	**β**	**95% CI**	***R^**2**^***	***p-value***	**β**	**95% CI**	***R^**2**^***	***p-value***
ILIβ	<−0.01	−0.003 to −0.001	0.10	<0.01	<−0.01	−0.001 to 0.001	0.02	0.81
IL2	−0.01	−0.167 to −0.028	0.09	<0.01	−0.02	−0.076 to 0.070	0.02	0.94
IL4	−0.48	−0.969 to 0.007	0.04	0.05	0.095	−0.401 to 0.592	0.02	0.70
IL6	<−0.01	−0.002 to 0.0003	0.09	<0.01	<−0.01	−0.002 to 0.000	0.17	<0.01
IL-10	−0.02	−0.058 to 0.013	0.02	0.21	0.01	−0.026 to 0.043	0.02	0.61
IL-12	−0.06	−0.188 to 0.065	0.01	0.34	0.02	−0.103 to 0.151	0.02	0.70
IL-13	−0.02	−0.066 to 0.019	0.01	0.28	0.02	−0.026 to 0.060	0.02	0.43
IFN-γ	−0.02	−0.025 to −0.005	0.09	<0.01	<−0.01	−0.011 to 0.010	0.02	0.90
TNFα	<−0.01	−0.004 to −0.001	0.09	<0.01	<−0.01	−0.004 to-0.001	0.17	<0.01

Unadjusted and adjusted linear regression β coefficients for breast milk cytokines predicting WAZ at final visit (*adjusted for maternal anemia).

*CI, Confidence interval; WAZ, Weight-for-age Z score; IL, interleukin; IFN, interferon; TNF, tumor necrosis factor; IGF, insulin-like growth factor*.

## Discussion

In many sub-Saharan African countries, infants are small at birth, show catch-up growth initially, but then rapidly demonstrate reduced growth velocity, leading to substantial growth faltering that becomes maximal by their second year of life (30, 33). Our findings show a similar pattern with clear growth faltering across the first 2–3 months postpartum. Identifying the factors that contribute to this pattern of growth—despite optimal feeding practices—is important, as developing interventions to optimize BM bioactive components could reduce the risk of poor growth and morbidity in early infancy, particularly in low-income settings (10, 13, 30, 31, 40). Using data from a cohort of mother infant pairs from urban Gambia, we have shown that variations in BM cytokine levels do not play a substantial role in the growth faltering observed across early infancy.

To date, there has been a paucity of studies in the literature reporting on cytokines and growth factors in BM and their role in growth and development in the early postnatal period. Previous studies have primarily focussed on weight gain, exploring the relationship between BM factors, particularly hormones, and infant adiposity and risk of obesity (13, 41–47). To our knowledge, we are the first to examine the relationship between BM cytokine profile and longitudinal growth outcomes, specifically WAZ, in the context of early growth faltering in a low-income setting.

The protective effects of bioactive factors in BM on infant health and developmental outcomes are thought to be mediated by immunomodulatory processes. More specifically, cytokines may play a role in enhancing sIgA production by neonatal leucocytes (e.g., TNFα, TGFβ2, IL6, IL10) (26, 48); in promoting infant gut maturity and barrier function (e.g., EGF, IGF1, TGFβ2) (23, 28, 40, 49); in boosting resistance to pathogen colonization (38); and in dampening inflammatory responses (e.g., IL2, IL10, TGFβ2) (6, 29, 48, 49). However, the exact mechanisms underlying growth patterns remain speculative. One hypothesis, proposed in the context of human milk oligosaccharides (HMOs), is that milk composition may shift toward a more protective profile, associated with a lower risk of infection and inflammation, thereby enabling the infant to invest energy in growth (7, 9, 10).

A cross-sectional preliminary study by Fields et al. suggested that BM concentrations of cytokines (IL6, TNF-α) and hormones/growth factors (insulin, glucose and leptin) may differentially influence weight gain and the development of fat and lean body mass in infants in the early postpartum period. WHO indicators of infant growth (Z scores), however, were not calculated or applied in their analysis (47). By contrast, our study focussed on an important WHO indicator of growth, WAZ, in the context of growth faltering; in our Gambian cohort, there was a decline in WAZ in over two-thirds of infants with 15% becoming underweight by 2–3 months postpartum, despite no infant being underweight at birth. Moreover, there was no significant correlation between colostrum cytokine levels and change in WAZ across the first 2–3 months post-partum, both in the adjusted and unadjusted models. The interrelationship of other colostrum factors, such as micronutrients or other bioactive analytes, including HMOs and hormones (adiponectin, insulin, ghrelin and leptin), may play a bigger role in variable growth outcomes and is currently under investigation (9–11, 13, 41–47, 50).

On the other hand, our findings suggest that cytokines in mature BM may predict infant growth measured at that same point in time. Higher levels of specific pro-inflammatory BM cytokines were negatively associated with WAZ at the final visit in our cohort, although the relationship was weak and clinical significance remains as yet unclear. The relationship between a pro-inflammatory environment and poor weight gain, both in the fetus and infant, has been previously described (46, 51–54). More specifically, the importance of TNFα and IL6 to early infant growth has already been suggested by Fields et al. (47). Higher concentrations of BM IL-6 were significantly associated with lower relative weight, weight gain, percent fat, and fat mass in healthy term infants at 1 month of age; higher TNF-α significantly correlated with lower lean mass but not measures of adiposity (47). A subsequent longitudinal study by the same group, however, could not replicate these findings, demonstrating only non-significant associations with infant body composition (46).

Cytokines in mature BM may impact infant growth through immunomodulation, as discussed earlier. Equally, these associations may represent context-specific correlations, rather than causation. For example, the cytokine profile in BM may reflect maternal health at this specific time point, including nutritional and disease status, which in turn may play a key role in growth outcomes. Further elucidation of factors that shape mature BM cytokine profile and understanding of underlying mechanistic or causational processes is therefore needed. This may have translational implications, such as the identification of maternal groups with high risk of underweight infants, and initiation of appropriate interventions subsequently.

Our study has several limitations. Firstly, it is a pilot exploration of outcomes in a small maternal-infant cohort within a limited time period. Given that (a) growth faltering is thought to start in the first few months of life, reaching its peak by 2 years of age in poorly-resourced settings and (b) cytokine profiles vary within and between mothers, it would be worth analyzing BM composition and growth outcomes in a larger cohort, at multiple time points and beyond 3 months postpartum. This would enable a more accurate WAZ trajectory to be plotted and the longer-term impact of BM cytokine differences to be fully appreciated. Of note, infants are thought to demonstrate a non-linear weight gain over this early post-partum period, and so weight at the final time point could either reflect a change in recent infant mass/weight or infant size more globally. Moreover, analysis of different types of BM and at various time points not evaluated in our study (e.g., foremilk vs. hindmilk; timing of sample collection in relation to feeding, lactational stage, or seasonal changes) may be valuable and has previously been shown to be important (4, 12, 15, 16, 55).

Furthermore, our focus was on one primary growth outcome: weight and associated WAZ scores. The latter is a well-established WHO growth indicator, especially at birth (when length measures may be difficult) and throughout the early postnatal period. Weight-for-length (WLZ) and length-for-age (LAZ) Z scores are equally important beyond the first year of life; stunting in early childhood has been shown to predict future risk of morbidity and mortality (32, 56) although is unlikely in the first 90 days post-partum. Similarly, head-circumference-for-age Z score (HAZ) is thought to be a good indicator of more chronic poor growth and undernutrition (57–59). Other potential growth outcomes to be measured include body composition factors (e.g., lean and fat mass, skinfold thickness), currently being explored particularly in BM hormone and obesity studies (11, 13, 41, 60).

In addition, our study eligibility criteria were strict, for example, infants born very prematurely or in poor condition requiring resuscitation (with subsequent transfer to neonatal intensive care) or women with high risk conditions in pregnancy were excluded. Future studies focussing on these high-risk groups may provide further insight into variables that regulate BM profile and, subsequently, boost or protect against growth faltering.

Finally, our study did not measure the contribution to early postpartum growth of other sources of immune factors, including the ante-and perinatal environment (cord blood, maternal non-milk derived cytokines) and endogenous postnatal production (infant serum). In parallel, the interplay of other bioactive factors, such as hormones and human milk oligosaccharides, must be explored.

In conclusion, our findings suggest that the interactions of cytokines in both colostrum and BM do not play a substantial role in the early growth faltering observed in Gambian infants. Above all, this preliminary study is valuable as a strong foundation to direct further investigation into the impact of bioactive factors in BM on infant growth and development, a topic with limited studies to date. Improved understanding of the early postnatal determinants of growth may be important for developing successful interventions to boost growth and health outcomes, particularly in infants at highest risk or in poorly resourced settings.

## Ethics Statement

All subjects gave informed consent in accordance with the Declaration of Helsinki. Each participant signed an informed consent form or, in case of illiteracy, thumb-printed and the consent form was signed by an impartial witness. The study was approved by and carried out in accordance with the recommendations of the joint Gambian Government/Medical Research Council Research Ethics Committee, SCC1350V4.

## Author Contributions

AS was responsible for manuscript writing and had input into the data analysis. DM, SM, and KL had input into the manuscript writing. AF was involved in laboratory analysis. OB was responsible for statistical analysis. KL was responsible for the original study design, data and statistical analysis and original idea for manuscript. All authors had access to the data and input into the final manuscript.

### Conflict of Interest Statement

KL has received funding from Pfizer to attend a seminar in 2016. The remaining authors declare that the research was conducted in the absence of any commercial or financial relationships that could be construed as a potential conflict of interest.

## Abbreviations

IL: Interleukin
IGF: Insulin-like growth factor
EGF: Epidermal growth factor
TGF: Transforming growth factor
HMO: Human milk oligosaccharide
Hb: Hemoglobin
BM: Breast milk
WAZ: Weight-for-age Z score
SD: Standard deviation
CI: Confidence interval.
