# Supplementary material for: Breast Milk Cytokines and Early Growth in Gambian Infants
Source: Front Pediatr. 2019 Jan 17;6:414. doi: 10.3389/fped.2018.00414 (PMC6344434; doi:10.3389/fped.2018.00414)
Supplement: Supplementary file 1 [file Data_Sheet_1.pdf]

Breast milk cytokines and early growth in Gambian infants

Supplementary Material

Table 1: Lowest Limit of Detection (LLOD) for cytokines measured using Meso Scale mcethods outlined in this study (Data from Meso Scale Discovery, 2014)

Cytokine	Lowest Limit of Detection
**IL-1β**	0.04
**IL-2**	0.09
**IL-4**	0.02
**IL-6**	0.06
**IL-10**	0.03
**IL-12p70**	0.11
**IL-13**	0.24
**TNFα**	0.04
**IFNγ**	0.2
**IGF-1**	26.8
**TGFβ2**	265.0

All cytokine measurements are in pg/ml

Table 2: Median of cytokine concentrations in colostrum and mature breast milk

Cytokine	Colostrum median (IQR)	Total samples	Number of samples detectable (%)*	BM median (IQR)	Total samples	Number of samples detectable (%)	p-value**	p-value for proportion of detectable samples***
**TNFα**	21.32 (7.27-104.59)	83	83 (100)	1.04 (0.55-2.49)	90	86 (96)	p<0.001	0.12
**IFNγ**	13.40 (5.87-29.93)	83	82 (99)	2.50 (0.83-5.96)	90	82 (91)	p<0.001	0.04
**IL10**	3.46 (1.11-12.47)	83	82 (99)	0.05 (0.02-0.23)	90	57 (63)	p<0.001	p<0.001
**IL12**	0.85 (0.21-3.45)	83	68 (82)	0.12 (0.06-0.28)	90	48 (53)	p<0.001	p<0.001
**IL13**	7.77 (1.82-24.67)	83	78 (94)	1.53 (0.59-2.94)	90	74 (82)	p<0.001	0.02
**IL1β**	6.00 (2.20-19.92)	83	83 (100)	0.40 (0.06-1.89)	90	71 (79)	p<0.001	p<0.001
**IL2**	0.84 (0.35-2.70)	83	78 (94)	0.11 (0.04-0.48)	90	52 (58)	p<0.001	p<0.001
**IL4**	0.16 (0.05-0.57)	83	72 (87)	0.01 (0.01-0.06)	90	40 (44)	p<0.001	p<0.001
**IL6**	25.91 (8.43-73.08)	83	83 (100)	0.91 (0.37-3.73)	90	85 (94)	p<0.001	0.06
**TGFβ2**	5116.49 (3464.15-8156.59	42	42 (100)	7983.20 (3842.67-19569.42)	46	46 (100)	0.11	1
**IGF-1********	13.40 (13.40-13.40)	70	15 (21)	13.40 (13.4-13.4)	90	10 (11)	0.06	0.08

All cytokine measurements are in pg/ml (rounded to 2 decimal places)

*Samples were termed as detectable if concentrations measured were above the lower limits of detection shown in Table 1.

**Wilcoxon rank sum test comparing the mean concentration of each cytokine in colostrum and breast milk

*** Fisher's exact two-sided test comparing proportions of detectable samples in colostrum and breast milk

****Median and IQR of IGF-1 concentrations in colostrum and BM samples coincide because the levels were below the assay limit of detection in >75% of subjects. Given this low detection rate, data on IGF-1 has been removed from further statistical analysis.

Abbreviations: BM, breast milk; SD, standard deviation; IQR, interquartile range

Table 3: Association between cytokine concentrations in colostrum and breast milk

**Cytokine**	***r****	***p*-value**
**TNFα**	0.07	0.57
**IFNγ**	0.06	0.61
**IL10**	0.14	0.21
**IL12**	0.26	0.02
**IL13**	0.04	0.73
**IL1β**	0.10	0.39
**IL2**	-0.08	0.46
**IL4**	0.14	0.21
**IL6**	0.14	0.22
**IGF-1**	0.21	0.08
**TGFβ2**	0.11	0.50

All values are rounded to 2 decimal places

* *r* = Spearman rank correlation co-efficient
